# Characterization of UGT8 as a monogalactosyl diacylglycerol synthase in mammals

**DOI:** 10.1093/jb/mvae084

**Published:** 2024-12-10

**Authors:** Yohsuke Ohba, Mizuki Motohashi, Makoto Arita

**Affiliations:** Division of Physiological Chemistry and Metabolism, Graduate School of Pharmaceutical Sciences, Keio University, 1-5-30 Shibakoen, Minato-ku, Tokyo 105-8512, Japan; Laboratory for Metabolomics, RIKEN Center for Integrative Medical Sciences (IMS), 1-7-22 Suehiro-cho, Tsurumi-ku, Yokohama, Kanagawa 230-0045, Japan; Division of Physiological Chemistry and Metabolism, Graduate School of Pharmaceutical Sciences, Keio University, 1-5-30 Shibakoen, Minato-ku, Tokyo 105-8512, Japan; Division of Physiological Chemistry and Metabolism, Graduate School of Pharmaceutical Sciences, Keio University, 1-5-30 Shibakoen, Minato-ku, Tokyo 105-8512, Japan; Laboratory for Metabolomics, RIKEN Center for Integrative Medical Sciences (IMS), 1-7-22 Suehiro-cho, Tsurumi-ku, Yokohama, Kanagawa 230-0045, Japan; Cellular and Molecular Epigenetics Laboratory, Graduate School of Medical Life Science, Yokohama City University, 1-7-22 Suehiro-cho, Tsurumi-ku, Yokohama, Kanagawa 230-0045, Japan; Human Biology-Microbiome-Quantum Research Center (WPI-Bio2Q), Keio University, 35 Shinanomachi, Shinjuku-ku, Tokyo 160-8582, Japan

**Keywords:** endoplasmic reticulum stress, glycolipid, mass spectrometry, membrane lipid, unfolded protein response

## Abstract

Monogalactosyl diacylglycerol (MGDG) is a major membrane lipid component in plants and is crucial for proper thylakoid functioning. However, MGDG in mammals has not received much attention, partly because of its relative scarcity in mammalian tissues. In addition, the biosynthetic pathway of MGDG in mammals has not been thoroughly analysed, although some reports have suggested that UGT8, a ceramide galactosyltransferase, has the potential to catalyse MGDG biosynthesis. Here, we successfully captured the endogenous levels of MGDG in HeLa cells using liquid chromatography quadrupole time-of-flight mass spectrometry (LC–QTOF-MS)-based lipidomics. Cellular MGDG was completely depleted in CRISPR/Cas9-mediated UGT8 knockout (KO) HeLa cells. Transient overexpression of UGT8 enhanced MGDG production in HeLa cells, and the corresponding cell lysates displayed MGDG biosynthetic activity *in vitro*. Site-directed mutagenesis revealed that His358 within the UGT signature sequence was important for its activity. UGT8 was localized in the endoplasmic reticulum and activation of the unfolded protein response by membrane lipid saturation was impaired in UGT8 KO cells. These results demonstrate that UGT8 is an MGDG synthase in mammals and that UGT8 regulates membrane lipid saturation signals in cells.

## Graphical Abstract

**Graphical Abstract:**
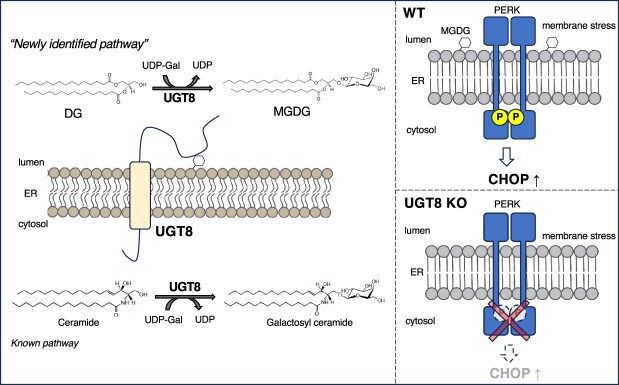

## Abbreviations

CIAP: alkaline phosphatase, calf intestinal
GalCer: galactosyl ceramide
GlcCer: glucosyl ceramide
HexCer: hexosyl ceramide
LC-QTOF-MS: liquid chromatography quadrupole time-of-flight mass spectrometry
MGDG: monogalactosyl diacylglycerol
UDP: uridine diphosphate
UGT: UDP-glycosyltransferase
UPR: unfolded protein response

Monogalactosyl diacylglycerol (MGDG) is the major membrane lipid component in the chloroplasts of photosynthetic cells, accounting for ~50% of the total lipid components *(**1**)*. This process is closely related to the biogenesis and function of the thylakoid membrane system in chloroplasts *(**2**)*. Cyanobacteria have a high abundance of MGDG, suggesting that MGDG is important for oxygenic photosynthetic organisms *(**3**)*. In plants, galactose is conjugated to diacylglycerol (DG) with a wide range of polyunsaturated fatty acids, and MGDG exhibits non-bilayer formation properties *(**4**)*. On the other hand, MGDG is a very minor lipid class in mammals, and has not received much attention until recently. In addition, MGDG synthases in *Arabidopsis thaliana* (MGD1, MGD2 and MGD3), which transfer galactose from UDP-galactose (UDP-Gal) to DG *(**2**,**5**)*, are not conserved in mammals. With progress in lipidomics technology, MGDG has been detected as a minor component of the mouse brain and kidney *(**6**,**7**)*.

UGT-glycosyltransferases (UGTs) are enzymes that catalyse the transfer of sugars from nucleotide UDP-sugars to a wide range of lipids and lipophilic molecules. In mammals, the UGT superfamily is categorized into four groups: UGT1, UGT2, UGT3 and UGT8. A total of 22 proteins belong to this superfamily, and UGT8 contains only one member, UGT8A1 (hereafter referred to as UGT8) *(**8**)*. Similar to other UGT proteins, UGT8 has a UGT domain structure and signature sequence with a single transmembrane domain *(**9**)*. Unlike other UGTs that use UDP-glucuronic acid (UGT1 and UGT2) or UDP-*N*-acetylglucosamine (UGT3) as substrates, UGT8 uses UDP-galactose as a sugar donor *(**10**)*. UGT8, initially named ceramide galactosyltransferase (CGT), synthesizes galactosylceramide (GalCer). GalCer is the main component of the myelin sheath and constitutes almost one-third of the lipid mass *(**11**)*. In the case of GalCer synthesis, a study using UGT8-knockout (KO) mice has confirmed UGT8 is the only enzyme for GalCer synthesis in the brain and is involved in myelin function and stability *(**12**,**13**)*. UGT8 has also been suggested to produce MGDG and galactosylated bile acid in UGT8-overexpressed cell lines *(**14**,**15**)*. However, gene knockdown or KO studies addressing these activities are lacking.

In this study, we successfully captured the endogenous level of MGDG in HeLa cells, and cellular MGDG was completely depleted in CRISPR/Cas9-mediated UGT8-KO HeLa cells. These results enabled us to characterize UGT8 as an MGDG synthase in mammals.

## Experimental Procedures

### Reagents

The UGT8 inhibitor (compound 19) and SCD1 inhibitor (A939572) were purchased from Cayman Chemicals. PNGase F was purchased from Thermo Fisher Scientific. Alkaline Phosphatase (Calf intestine) (CIAP) was purchased from Takara. UDP-Galactose and UDP-Glucose were purchased from Sigma-Aldrich. Tunicamycin was purchased from Fujifilm (Wako Pure Chemical Industries). The DG (10:0/10:0) and C2 Ceramide (d18:1/2:0) were purchased from Avanti Polar Lipids. The following commercially available antibodies were used: UGT8 (Sigma-Aldrich, Cat#HPA014405), GAPDH (Merck, Cat#CB1001), FLAG (Sigma Aldrich, Cat#F1804), PERK (Cell Signaling Technology, Cat#3192), calnexin (Abcam, Cat#ab22595), Tom20 (Cell Signaling Technology, #Cat42406), LAMP1 (Cell Signaling Technology, Cat#9091), calreticulin (Cell Signaling Technology, Cat#12238), VDAC1 (Abcam, Cat#ab14734) and actin (Sigma-Aldrich, Cat#A2066).

### Cell culture, transfection and RNA interference

HeLa cells and HEK293T cells (ATCC) were maintained in Dulbecco’s modified Eagle’s medium (DMEM) supplemented with L-glutamine, 10% foetal bovine serum penicillin (100 μg/ml), and streptomycin (100 μg/ml). Cell lines were maintained at 37 °C and 5% CO_2_ and were routinely tested for mycoplasma infections. Cells were transfected with DNA expression vectors using ViaFect (Promega). Lipofectamine RNAiMax (Invitrogen) was used for siRNA transfection. Silencer Select Negative Control siRNA (Ambion, Cat#4390843) and Silencer Select predesigned siRNAs against UGT8 (Ambion, cat #s14663, s14664, and s14665) were used.

### Plasmid construction

Complementary DNA (cDNA)-encoding mouse Ugt8 with an N- or C-terminal FLAG tag and human UGT8 with a C-terminal FLAG tag were cloned into the pCAGGS or pLJC5 (Addgene) vector. The cDNA-encoding mutant mUgt8 (H358A or H383Q) was generated using site-directed mutagenesis polymerase chain reaction (PCR).

### Generation of KO cell lines

HeLa cells lacking UGT8 were generated using CRISPR/Cas9-mediated gene editing. The sequence AACTCTGGGACGTATGCTAA was used as the gRNA. Briefly, gene-specific DNA fragments were synthesized, cloned into pX459 (Addgene) and transfected into cells. Transfected cells were selected for using puromycin (1 μg/ml). Single clones were picked by limiting dilution, and mutations were confirmed by genomic sequencing.

### Generation of stable expression cell lines

For lentiviral infection, HEK293T cells were transiently transfected with pLJC5 (containing the gene of interest) for 48 h using Lenti-X packaging single shoots (VSV-G) (Takara). Virus-containing culture supernatants were collected and cleared through a 0.45-μm filter before being added to UGT8 KO HeLa cells in the presence of 4 μg/ml Polybrene. Infected cells were selected for using puromycin (1 μg/ml).

### Immunoblotting

HeLa cells were washed with ice-cold phosphate-buffered saline (PBS) and lysed with RIPA buffer (50 mM Tris/HCl pH 7.4, 150 mM NaCl, 1 mM EDTA, 1% (v/v) Triton X-100, 0.1% (w/v) SDS and 0.5% (w/v) Na-deoxycholate) containing cOmplete EDTA free (Roche) and PhosSTOP (Roche) for 30 min. After centrifugation at 15,000 rpm for 10 min at 4°C, supernatant fractions were collected and analysed using sodium dodecyl sulfate-polyacrylamide gel electrophoresis (SDS-PAGE) and immunoblotting. For Phos-tag SDS-PAGE, 25 μM Phos-tag AAL-107 (Fujifilm Wako) and 50 μM MnCl_2_ containing acrylamide-gel was used for protein separation.

### Immunofluorescence

HeLa cells grown on glass coverslips were fixed with 4% (v/v) paraformaldehyde in PBS for 15 min, permeabilized with 0.1% (v/v) Triton X-100 for 10 min and incubated with primary antibodies in PBS containing 3% (w/v) bovine serum albumin. After washing, the cells were incubated with Alexa Fluor-conjugated secondary antibody. After washing, coverslips were mounted onto slides using ProLong Gold (Thermo Fisher Scientific) and imaged using a confocal fluorescence microscope (FV3000; Olympus).

### Quantitative real-time PCR

Total RNA was extracted from HeLa cells using ISOGEN II (NIPPON GENE), and reverse transcription was performed using ReverTra Ace qPCR RT Master Mix (TOYOBO). Quantitative real-time PCR was performed using the THUNDERBIRD SYBR qPCR Mix (TOYOBO). The CHOP expression level was normalized to that of GAPDH. The primer sequences were as follows:

CHOP fw: 5’-AGCTGGAAGCCTGGTATGAG- 3’.

CHOP rv: 5’-GTGCGTGTGACCTCTGTTGG- 3’.

GAPDH fw: 5’-GAACGGGAAGCTCACTGGCATGGCC- 3’.

GAPDH rv: 5’-TGTCATACCAGGAAATGAGCTTGAC- 3’.

### *In vitro* galactosyltransferase or glucosyltransferase activity assay

*In vitro* galactosyltransferase or glucosyltransferase activity assay was performed as previously described *(**14**)*. Briefly, 10 μg of cell lysates were incubated for 1 h at 37°C in a buffer (120 mM potassium glutamate,15 mM KCl, 5 mM NaCl, 0.8 mM CaCl_2_, 2 mM MgCl_2_, 2 mM MnCl_2_, 1.6 mM EGTA and 20 mM Hepes-KOH, pH 7.4) containing 1 mM UDP-Gal or UDP-Glc, and 20 μM DG (10:0/10:0) or C2 ceramide. The reaction was terminated by the addition of methanol.

### Microsomal fractionation

Collected HeLa cells were resuspended in homogenization buffer (220 mM mannitol, 70 mM sucrose, 5 mM HEPES/KOH pH 7.4, 1 mM EGTA) containing cOmplete EDTA free (Roche), homogenized using a 23 G syringe (Becton Dickinson) (15 strokes) and centrifugated at 600 *g* for 5 min. The resulting supernatant was further centrifuged at 8,000 *g* for 10 min at 4°C and collected the supernatant. The ultracentrifugation (100,000 *g*, 30 min, 4°C) was performed with the supernatant and the resulting pellet was used as microsome fraction.

### Untargeted lipidomics

Total lipids were extracted using a single-phase extraction method with a mixed solvent (MeOH:CHCl_3_:H_2_O, 2:1:0.2, v/v/v) as previously described *(**16**)*. Briefly, 1 million cells, 20 μg protein of microsomal fraction or a part of assay mixture were mixed with 200 μl of MeOH containing 5 μl of EquiSPLASH (Avanti Polar Lipids) and incubated for 1 h at room temperature. One hundred microlitres of CHCl_3_ was added, and the samples were further incubated for 1 h. Finally, 20 μl of Milli-Q water was added and incubated for 10 min. After extraction, the samples were centrifuged at 2,000 × *g* for 10 min, and the supernatants were collected for lipidomic analysis. For the measurement of the *in vitro* assay products, MGDG (18:1/18:1) and C18 Galactosyl(ß) Ceramide (d18:1/18:0) (Avanti Polar Lipids) were added as internal standards. Extracted lipids were analysed using an LC-QTOF/MS system (LCMS-9030, Shimadzu) operated in data-dependent acquisition (DDA) mode, as described previously *(**17**)*. LC separation was performed using an ACQUITY UPLC Peptide BEH C18 (2.1 × 50 mm, Waters) with mobile phase A (methanol: acetonitrile: water = 1:1:3, v/v/v, containing 5 mM ammonium acetate and 10 nM EDTA) and B (2-propanol containing 5 mM ammonium acetate and 10 nM EDTA). The flow rate was 0.3 ml/min and the column was kept at 45°C. LC gradient consisted of solvent A for 0.5 min, then linearly converting to solvent (A:B = 60:40) for 4 min, linearly converting to solvent (A:B = 36:64) for 2.5 min and holding for 4.5 min then linearly converting to solvent (A:B = 17.5/82.5) for 0.5 min, linearly converting to solvent (A:B = 15:85) for 6.5 min and linearly converting to solvent (A:B = 5:95) for 1 min followed by returning to solvent A and holding for 5 min. The parameters for DDA were identical to those reported previously *(**17**)*; MS1 and MS2 start-end time: 0.5–18.5 min; MS1 and MS2 mass ranges: m/z 70–1750; collision energy: 35 eV; collision energy spread: 20 eV; MS1 event time: 250 ms; MS2 event time: 66 ms; interface voltage: 4.00 kV (+)/−3.00 kV (−); number of dependent events: 15; CID gas: argon, 230 kPa; ionization mode: ESI; nebulizer gas: 2.0 l/min; heating gas: 20.0 l/min (+)/10.0 l/min (−); interface temperature: 100°C (+)/300°C (−); drying gas: 10.0 l/min; DL temperature: 250°C (+)/300°C (−); and heat block temperature: 400°C. The obtained data were processed using MS-DIAL version 5.1 *(**18**)*. The analysis parameters were identical to those reported previously *(**16**)* except for the following: minimum peak height, 1,000; accurate mass tolerance (MS2), 0.025 Da; and retention time tolerance for alignment: 0.1 min. Concentrations of EquiSPLASH-containing lipid classes were calculated from the peak area of the precursor ion of deuterated standards of each lipid in EquiSPLASH. The concentrations of other lipid classes were calculated from the peak areas of the precursor ions of the deuterated standards of lysophosphatidylcholine in EquiSPLASH. For the calculation of the *in vitro* assay products, the peak areas of MGDG (18:1/18:1) and C18 Galactosyl(ß) Ceramide (d18:1/18:0) were used to calculate the *in vitro* assay products.

### Statistical analyses

Statistical significance was assessed using a two-tailed *t*-test or one-way analysis of variance (ANOVA) as indicated. All reported numbers refer to biological replicates. Statistical significance was set at *P* < 0.05.

**Fig. 1:**
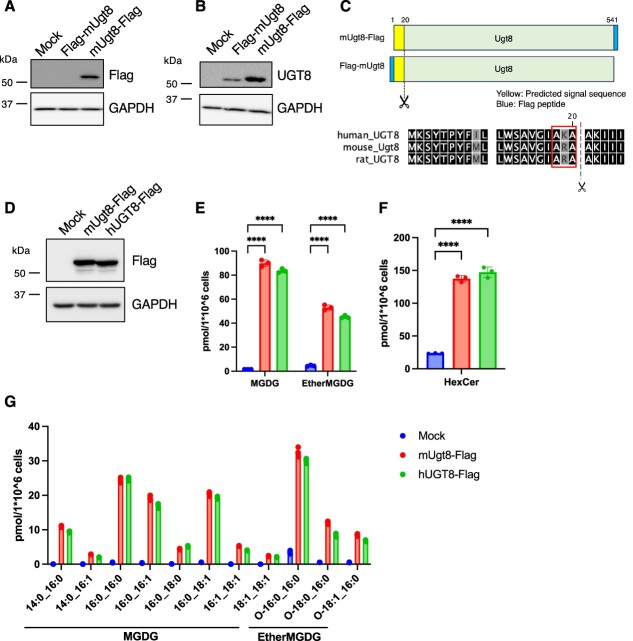
**Exogenous expression of mouse and human UGT8 increased the MGDG content in HeLa cells.** (A, B) Immunoblot analysis of HeLa cells transiently expressing N- or C-terminally FLAG-tagged mouse Ugt8. The indicated antibodies were used. (C) The predicted cleavage site of UGT8 (upper panel). AXA (Alanine—X—Alanine) is a canonical motif recognized by signal peptidase, which is conserved in human, mouse and rat UGT8 (lower). (D) Immunoblot analysis of HeLa cells transiently expressing C-terminal FLAG-tagged mouse or human UGT8. Indicated antibodies were used. (E–G) Lipid analysis of HeLa cells transiently expressing C-terminally FLAG-tagged mouse or human UGT8. The MGDG (E) and HexCer (F) contents and the fatty acid composition of MGDG (G) are shown (*n* = 3). Mean ± SD. ^****^P < 0.0001, one-way ANOVA with Dunnett’s multiple comparisons test (E–G).

## Results

### UGT8 overexpression increased MGDG content in HeLa cells

It has been demonstrated that UGT8 synthesizes MGDG in CHO cells overexpressing rat UGT8 *(**14**)*. We first confirmed the effect of overexpressing mouse and human UGT8 in cultured human cell lines. N- or C-terminal FLAG-tagged mouse Ugt8 (FLAG-mUgt8 or mUgt8-FLAG) were transiently overexpressed in HeLa cells; however, only mUgt8-FLAG was successfully detected using anti-FLAG antibodies (Fig. 1A). When anti-UGT8 antibodies were used, both FLAG-mUgt8 and mUgt8-FLAG were detected; however, the size of FLAG-mUgt8 was slightly smaller than that of mUgt8-FLAG (Fig. 1B). These results indicate that mUgt8 was cleaved around the N-terminal region (Fig. 1C). In fact, mUgt8 was predicted to be cleaved between the 20th and 21st amino acid residues (Fig. 1C); these regions are conserved amongst rat, mouse and human UGT8, although these cleavages have not been previously determined. Hereafter, we used C-terminal-tagged UGT8 for analysis in order to detect the overexpressed proteins by anti-FLAG antibodies. Next, we analysed the lipid profiles of either mUgt8-FLAG or human UGT8 (hUGT8)-FLAG-overexpressing HeLa cells using LC-QTOF-MS. The levels of MGDG (galactose-conjugated diacylglycerol) and ether-linked MGDG (galactose-conjugated alkylacylglycerol) were significantly increased by UGT8 overexpression (Fig. 1D and 1E). Interestingly, MGDG and ether-linked MGDG mainly contained DG with saturated fatty acids (Fig. 1G). The amount of hexose-conjugated ceramide (HexCer) was also increased by the expression of UGT8 (Fig. 1F), indicating that the C-terminal FLAG-tagged UGT8 proteins function as ceramide galactosyltransferases. In addition, considering that the expression levels of mUgt8-FLAG and hUGT8-FLAG (Fig. 1D) and the increased amounts of MGDG and HexCer were comparable (Fig. 1E and 1F), mouse and human UGT8 proteins appeared to have similar enzymatic activities. Moreover, certain amounts of MGDG and ether MGDG were detected in mock-transfected control HeLa cells (Fig. 1E and 1G), indicating that MGDGs were endogenously produced in HeLa cells. Since MGDG was reported to exist in mouse kidney *(**7**)*, we compared the amount of MGDG between HeLa cells and kidney-derived cell line HEK293T cells, and found ether MGDG was more abundant in HeLa cells than HEK293T cells (Supplementary Fig. S1A). From these results, we decided to use HeLa cells for further analysis.

### UGT8 knockdown/inhibition decreased the amount of endogenous MGDG in HeLa cells

**Fig. 2:**
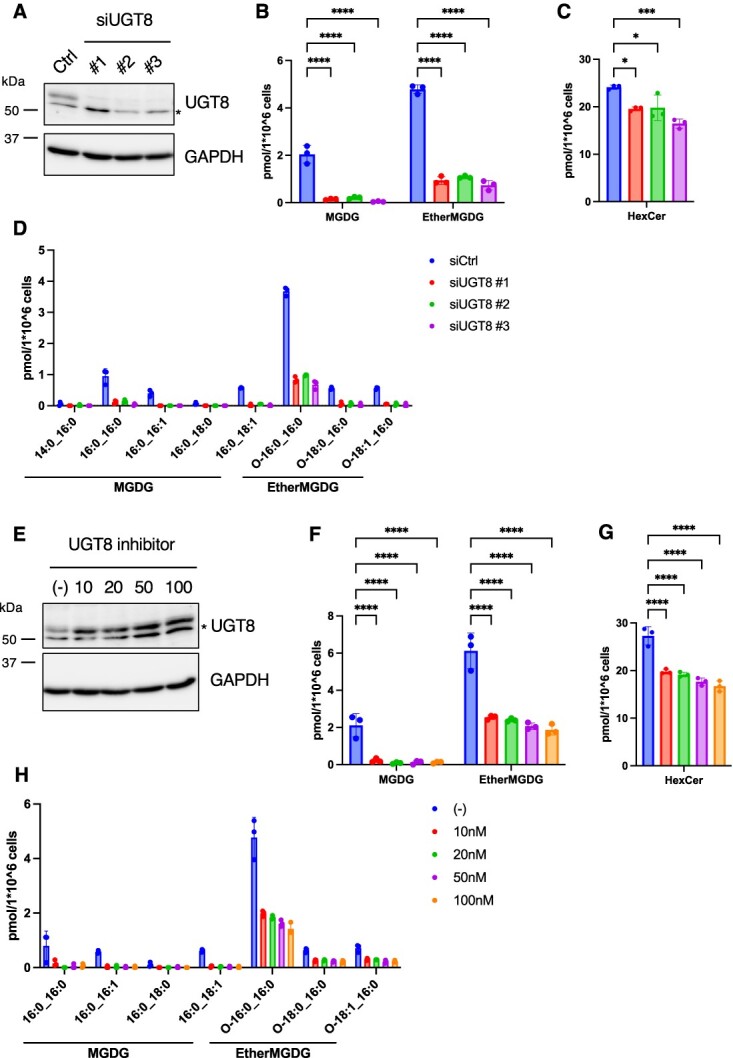
**UGT8 inhibition decreased the MDGD content in HeLa cells.** (A) Immunoblot analysis of HeLa cells treated with control or UGT8 siRNA for 72 h. Indicated antibodies were used. (B–D) Lipid analysis of HeLa cells treated with control or UGT8 siRNA for 72 h. The MGDG (B) and HexCer (C) contents and fatty acid composition of MGDG (D) are shown (*n* = 3). (E) Immunoblot analysis of HeLa cells treated with indicated concentrations of UGT8 inhibitor (compound 19) for 24 h. Indicated antibodies were used. (F–H) Lipid analysis of HeLa cells treated with indicated concentrations of UGT8 inhibitor (compound 19) for 24 h. The MGDG (F) and HexCer (G) contents and fatty acid composition of MGDG (H) are shown (*n* = 3). Mean ± SD. ^*^P < 0.05, ^***^P < 0.001, ^****^P < 0.0001, one-way ANOVA with Dunnett’s multiple comparisons test (B, C, F and G). ^*^ Cross reaction.

To determine whether endogenous MGDG production in HeLa cells was dependent on UGT8, we performed siRNA-mediated gene silencing of *UGT8*. We used three different siRNAs and confirmed that they all effectively suppressed the protein expression level of UGT8 (Fig. 2A). In UGT8-knockdown cells, the MGDG content was significantly decreased, although a certain amount of MGDG was still detected in these cells (92.8% reduction in MDGD and 80.7% reduction in ether MGDG on average in three different siRNA-treated cells) (Fig. 2B and 2D). In HeLa cells, both MGDG and ether-linked MGDG contained DG with C16:0 chains, and ether-linked MGDG (O-16:0_16:0) was the predominant form (Fig. 2D). On the other hand, the level of DG was higher than that of ether-linked DG (Supplementary Fig. S1B). Furthermore, ether-linked DG (O-16:0_16:0) was not the predominant form amongst DGs (Supplementary Fig. S1C). These results indicate that UGT8 preferentially utilize ether-linked DG (O-16:0_16:0) as a substrate. In contrast, the reduction in HexCer was relatively slight (22.9% average reduction in three different siRNA-treated cells) and HexCer (18:1; O2/24:0; O) was selectively reduced (Fig. 2C and Supplementary Fig. S1D), suggesting the presence of glucosyl ceramide (GlcCer) in HeLa cells.

Compound 19 is a commercially available UGT8 inhibitor (N-[(1S)-1-methyl-2-(trifluoromethoxy) ethyl]-carbamic acid, 1-[3-[(methylamino) carbonyl]-7-(trifluoromethyl) thieno [3, 2-b] pyridin-5-yl]-4-piperidinyl ester) *(**19**)*. Although it has not been proven whether the compound directly binds to UGT8 to inhibit its enzymatic activity, compound 19 has been shown to decrease the amount of GalCer in the adenocarcinoma cell line OE19 and the mouse brain and kidney *(**7**,**19**)*. After treating HeLa cells with various concentrations of the inhibitor, we found that the MGDG content decreased without suppressing UGT8 protein expression (Fig. 2E, 2F and 2H). Similar to UGT8 siRNA-treated cells, the reduction in HexCer levels by inhibitor treatment was relatively minor (Fig. 2G). These results indicated that UGT8 contributes to the endogenous production of MGDG in HeLa cells.

### MGDG was undetectable in UGT8 KO HeLa cells

**Fig. 3:**
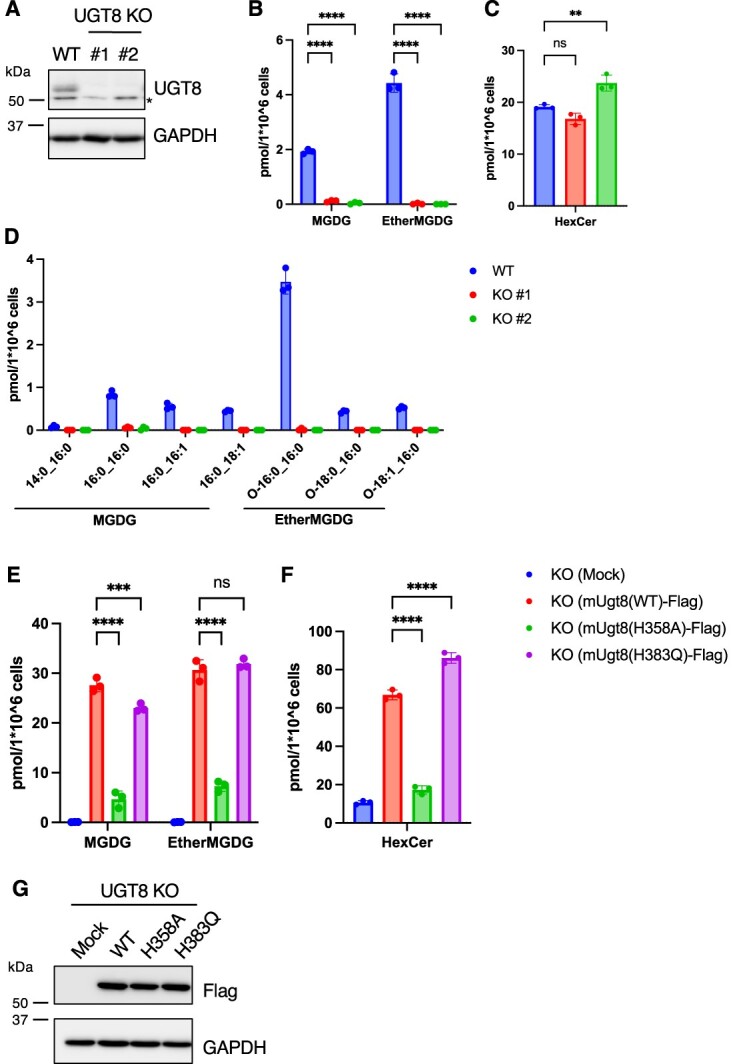
**Cellular MGDG was absent in UGT8 KO HeLa cells.** (A) Immunoblot analysis of WT or UGT8 KO HeLa cells. Indicated antibodies were used. (B–D) Lipid analysis of WT or UGT8 KO HeLa cells. The MGDG (B) and HexCer (C) contents and fatty acid composition of MGDG (D) are shown (*n* = 3). (E, F) Lipid analysis of UGT8 KO HeLa cells (#1) transiently expressing C-terminal FLAG-tagged mouse UGT8 (WT, H358A or H383Q). The MGDG (E) and HexCer (F) contents are shown (*n* = 3). (G) Immunoblot analysis of UGT8 KO HeLa cells (#1) transiently expressing C-terminal FLAG-tagged mouse UGT8 (WT, H358A or H383Q). Indicated antibodies were used. Mean ± SD. ^**^P < 0.01, ^***^P < 0.001, ^****^P < 0.0001, ns; not significant, one-way ANOVA with Dunnett’s multiple comparisons test (B, C, E and F). ^*^ Cross reaction.

Since a certain amount of MGDG remained in UGT8 siRNA-treated or UGT8 inhibitor-treated HeLa cells (Fig. 2B and 2F), we generated UGT8 KO cells to determine whether the production of MGDG was completely dependent on UGT8. We generated UGT8 KO HeLa cells using CRISPR/Cas9 and isolated two independent clones, as determined using immunoblotting (Fig. 3A). In these cells, both MGDG and ether-linked MGDG were undetectable, indicating that UGT8 is the only protein responsible for MGDG production in HeLa cells (Fig. 3B and 3D). In contrast, the amount of HexCer was maintained in the UGT8 KO cells (Fig. 3C) and the reduction of HexCer (18:1; O2/24:0; O) was milder than that of UGT8 siRNA-treated cells (Supplementary Fig. S1D and S1E), raising a possibility that the production of GlcCer increases as a compensation by complete depletion of UGT8.

**Fig. 4:**
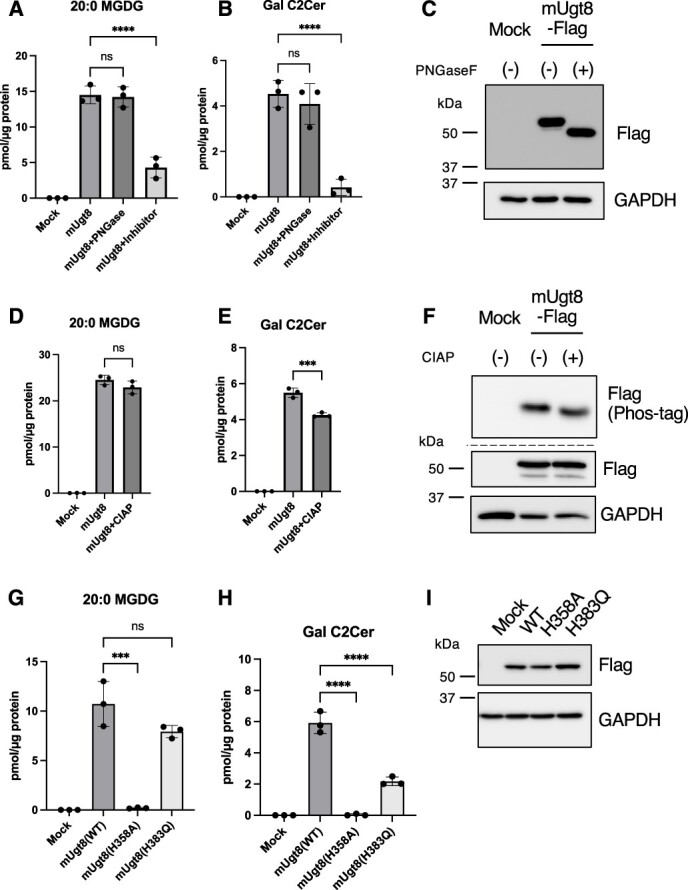
**Characterization of the enzymatic activity of UGT8 *in vitro.*** (A–F) Galactosyltransferase activity assay was conducted by incubating 1 mM UDP-Gal and 20 μM DG (10:0/10:0) (A and D) or C2 Ceramide (C2Cer) (B and E) in the presence of 10 μg of HeLa cell lysates expressing mUGT8 with or without UGT8 inhibitor (10 nM) for 1 h at 37°C (*n* = 3). For PNGase or CIAP treatment, mUGT8-overexpressed HeLa cell lysates were incubated with PNGase or CIAP for 30 min at 37°C prior to the assay. Exogenous expressions of UGT8 and the effect of PNGase or CIAP treatment were confirmed using either normal or phos-tag SDS-PAGE and immunoblotting (C and F). (G–I) Galactosyltransferase activity assay was conducted by incubating 1 mM UDP-Gal and 20 μM DG (10:0/10:0) (G) or C2 Ceramide (C2Cer) (H) in the presence of 10 μg of UGT8 KO (#1) HeLa cell lysates expressing indicated mUGT8 proteins (*n* = 3). Exogenous expressions of UGT8 proteins were confirmed using immunoblotting (I). Mean ± SD. ^***^P < 0.001, ^****^P < 0.0001, ns; not significant, two-tailed unpaired *t*-test (D, E), one-way ANOVA with Dunnett’s multiple comparisons test (A, B, G, H).

Amongst the UGT family of proteins, UGT8 is the only protein that uses UDP-Gal as a substrate. Histidine358 (H358) is a conserved amino acid residue within the UGT signature sequence in UGT family proteins and has been proposed to bind UDP-sugar *(**20**)*. In contrast, all mammalian UGTs, except UGT8, have aspartate and glutamine (DQ) motifs at the end of the UGT signature sequence *(**20**)*. In UGT8, glutamine is replaced by histidine (H383), which is proposed to be important for galactose recognition because the DH motif is conserved in plant UGTs that use UDP-galactose as a substrate *(**21**)*. We determined the importance of these residues (H358 and H383) in UGT8 activity by expressing the H358A or H383Q mutants in HeLa cells. Compared to wild-type (WT) mUgt8 expression, H358A mutant expression did not enhance MGDG or HexCer production, although the exogenous protein expression levels were comparable (Fig. 3E–3G). In contrast, H383Q mutants still had the ability to increase MGDG and HexCer contents, similar to WT mUgt8 (Fig. 3E–3G). These results indicated that H358 is an important residue in UGT8 for MGDG production, whereas the DH motif in UGT8 is not critical for the recognition of UDP-galactose.

### UGT8 has a UGT activity to DG *in vitro*

The cell-based experiments described above suggested that UGT8 is a potential MGDG synthase. To detect the UGT8-dependent enzymatic activity *in vitro*, HeLa cell lysates were used as enzymatic sources. We incubated UDP-Gal and DG (10:0/10:0) or C2 Ceramide (d18:1/2:0) with lysates of either mock- or mUgt8-FLAG-overexpressed HeLa cells and measured the production of galactose-transferred DG (10:0/10:0) or C2 Ceramide (d18:1/2:0) using LC-QTOF-MS. Although the production of MGDG (10:0/10:0) and GalCer (d18:1/2:0) was not detected in mock-transfected HeLa cell lysates, it was detected in mUgt8-FLAG-overexpressed HeLa cell lysates (Fig. 4A–4C). These activities were significantly suppressed when the UGT8 inhibitor was added to the reaction mixture. These results indicate that UGT8 is an enzyme that produces MGDG from DG and UDP-Gal. On the other hand, when UDP-Glc was used as a substrate instead of UDP-Gal in the assay, either monoglucosylDG (MGlcDG) or GlcCer was not producing UGT8 dependently, indicating that UGT8 specifically used UDP-Gal to produce galactose-conjugated lipids (SupplementaryFig. S2A and S2B). Next, we performed the assay with different amount of lipid substrates and found that DG was a better substrate than ceramide for UGT8 activity *in vitro* (Supplementary Fig. S2C). Glycosidase pretreatment of lysates did not affect UGT8 activity (Fig. 4A–4C), indicating that glycosylation of the UGT8 protein *(**9**)* is not essential for its enzymatic activity. Because proteomic studies have identified phosphorylated UGT8 peptides *(**22**,**23**)*, we monitored whether UGT8 was phosphorylated. mUgt8-FLAG-overexpressed HeLa cell lysates were incubated with or without calf intestinal alkaline phosphatase (CIAP) and separated using Phos-tag SDS-PAGE. CIAP treatment caused a size shift of the mUGT8-FLAG protein, suggesting that mUGT8-FLAG was phosphorylated (Fig. 4F). However, CIAP-treated mUgt8 cells displayed similar UGT activity to DG *in vitro*, indicating that phosphorylation was not involved in the regulation of UGT8 enzyme activity for MGDG production (Fig. 4D). On the other hand, CIAP treatment slightly but significantly reduced the production of GalCer raising a possibility that phosphorylation of UGT8 is partly involved in the regulation of GalCer synthesis (Fig. 4E). We evaluated the importance of H358 and H383 in UGT8 cells using *in vitro* assay*s.* UGT8 activity towards either DG or ceramide was not detected when mUgt8(H358A)-FLAG-overexpressing cell lysates were used, indicating the importance of H358 in its enzymatic activity (Fig. 4G–4I), which was consistent with the results of the cell-based assay (Fig. 3E–3G). Lysates of mUgt8(H383Q)-FLAG-overexpressing cells also exhibited UGT activity, indicating that H383 was not essential for UGT activity (Fig. 4G–4I).

**Fig. 5:**
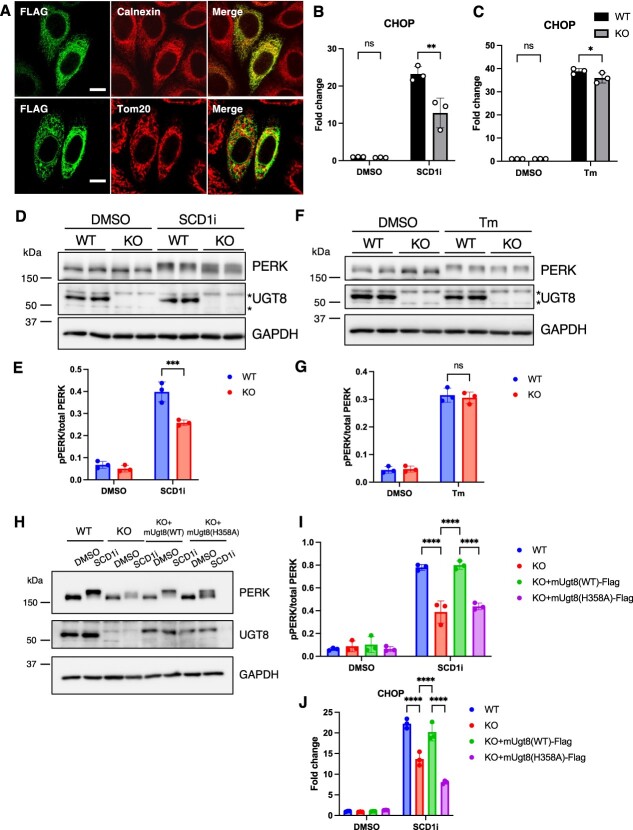
**Membrane lipid saturation-induced UPR was suppressed in UGT8 KO HeLa cells.** (A) Immunofluorescence of HeLa cells transiently expressing C-terminal FLAG-tagged mUgt8. Cells were immunostained with indicated antibodies. Scale bar, 10 μm. (B, C) The mRNA level of CHOP in WT or UGT8 KO (#1) HeLa cells treated with SCD1 inhibitor (100 nM) for 24 h (B) or tunicamycin (2.5 μg/ml) for 6 h (C) was quantified using qRT-PCR (*n* = 3). (D–G) PERK phosphorylation of WT or UGT8 KO (#1) HeLa cells treated with SCD1 inhibitor (100 nM) for 24 h (D, E) or tunicamycin (2.5 μg/ml) for 6 h (F, G) were evaluated using immunoblotting (representative data of *n* = 2 out of *n* = 3 independent experiments). The ratio of phosphorylated PERK (pPERK) to total PERK was quantified (E, G) (*n* = 3). (H–J) WT, UGT8 KO (#1) and UGT8 KO (#1) stably expressing mUgt8(WT)-FLAG or mUgt8(H358A)-FLAG HeLa cells were treated with SCD1 inhibitor (100 nM) for 24 h. PERK phosphorylation was evaluated using immunoblotting (F) (representative data of *n* = 2 out of *n* = 3 independent experiments). The ratio of phosphorylated PERK (pPERK) to total PERK was quantified (I) (*n* = 3). The mRNA level of CHOP was quantified using qRT-PCR (J) (*n* = 3). Mean ± SD. ^*^P < 0.05, ^***^P < 0.001, ^****^P < 0.0001, ns; not significant, two-tailed unpaired *t*-test (B, C, E, G), one-way ANOVA with Dunnett’s multiple comparisons test (I, J).

### Membrane lipid saturation-induced UPR was suppressed in UGT8 KO HeLa cells

To determine the potential functions of UGT8 and its products, we first determined the intracellular localization of UGT8. As reported previously *(**9**)*, overexpressed mUGT8-FLAG co-localized with the endoplasmic reticulum (ER) protein calnexin but not with the mitochondrial protein Tom20 (Fig. 5A). Next, we isolated microsomal fraction from HeLa cells and performed lipidomics. The ratio of MGDGs/PC was similar between microsomal fraction and whole cell, but the ratio of HexCer/PC was lower in microsomal fraction compared to whole cell (Supplementary Fig. S3A–S3C). These results indicate that MGDG is more enriched in the microsomal compartment compared to HexCer. Recently, it was reported that glycoprotein glucosyltransferase 2 (UGGT2) transfers glucose to phosphatidic acid with saturated fatty acids, and UGGT2 protects against ER stress caused by membrane lipid saturation *(**24**)*. Because most MGDG in HeLa cells contain saturated fatty acids (Fig. 3D), we investigated the relationship between UGT8 and membrane lipid saturation-induced ER stress. The inhibition of stearoyl-CoA desaturase 1 (SCD1) induces ER stress via membrane lipid saturation *(**25**)*. Treatment with the SCD1 inhibitor A939572 *(**26**)* caused membrane lipid saturation by increasing the ratio of saturated fatty acids/monounsaturated fatty acids in phosphatidylcholine (PC), a dominant phospholipid class in the cell membrane (Supplementary Fig. S3D) and induced membrane stress. Membrane stress activated the unfolded protein response (UPR), which was assessed by the induction of PKR-like ER kinase (PERK) phosphorylation and the upregulation of C/EBP homologous protein (CHOP) mRNA (Fig. 5B, 5D and 5E) *(**27**)*. PERK phosphorylation was visualized as a band shift of PERK to a higher molecular weight (Fig. 5D). Treatment with an SCD1 inhibitor did not significantly increase the amount of MGDG O-16:0_16:0, the dominant MGDG molecule (Supplementary Fig. S3E). In UGT8 KO cells, membrane lipid saturation-induced PERK phosphorylation and CHOP mRNA upregulation were significantly suppressed (Fig. 5B, 5D–E, and Supplementary Fig. S3F and S3G). Tunicamycin is an inhibitor of protein glycosylation that triggers ER stress by accumulating unfolded proteins in the ER *(**28**)*. When ER stress was induced by tunicamycin treatment, PERK phosphorylation and CHOP upregulation occurred to almost the same extent in WT and UGT8 KO cells (Fig. 5C, 5F and 5G). Tunicamycin treatment did not affect the concentration of MGDG O-16:0_16:0 (Supplementary Fig. S3E). Impaired lipid saturation-induced UPR in UGT8 KO cells was complemented by mUgt8(WT)-FLAG but not by mUgt8(H358A)-FLAG stable expression (Fig. 5H–5J). In mUgt8(WT)-FLAG-expressing cells, both MGDG and HexCer levels were increased (Supplementary Fig. S3H and S3I). These results indicate that UGT8 activity is required for proper activation of the UPR by membrane lipid saturation.

## Discussion

In this study, we used LC–MS/MS-based lipidomics to detect MGDG, a minor lipid class in mammals, and found that MGDG was present in HeLa cells. Although some reports have suggested a potential role for UGT8 in MGDG production *(**7**,**14**)*, a knockdown/KO approach to clarify the contribution of UGT8 to MGDG production has not yet been conducted. We detected the endogenous production of MGDG in HeLa cells and confirmed that its production was completely dependent on UGT8 using the CRISPR/Cas9-mediated gene KO technique. Furthermore, *in vitro* enzymatic assay showed that UGT8 had UDP-Gal but not UDG-Glc transferase activity to DG. These results suggest that MGDG production is completely dependent on UGT8 in HeLa cells. In addition, exogenous expression of N- or C-terminal epitope-tagged UGT8 demonstrated N-terminal cleavage of UGT8, consistent with the predicted signal peptide cleavage site *(**29**)*. Although DG with C16:0 chains are not predominant molecules amongst DG in HeLa cell, most MGDG contained DG with C16:0 chains in the cells. UGT8 uses ceramide as a substrate for GalCer synthesis. The structure of DG with C16:0 chains is similar to that of ceramide *(**30**)*; therefore, UGT8 may accept these ceramide-like DG as substrates. In fact, other than UGT8, sphingomyelin synthase uses both ceramide and DG as substrates *(**31**)*. Our *in vitro* assay showed DG was a better substrate than ceramide for UGT8 activity. However, the amount of MGDG and HexCer in HeLa cells were comparable, raising a possibility that ceramide is more accessible to UGT8 in HeLa cells.

To date, the regulation of the enzymatic activity of UGT8 has not been analysed. It has been reported that UGT8 undergoes N-linked glycosylation *(**9**)*. We also observed that the expressed UGT8 was deglycosylated by treatment with the recombinant glycosidase PNGase F, which cleaves N-linked oligosaccharides. However, PNGase F treatment did not affect the *in vitro* galactosyltransferase activity of UGT8, indicating that N-linked glycosylation was not involved in its enzymatic activity. Proteomics have identified phosphorylated UGT8 peptides *(**22**,**23**)*; however, a detailed analysis of UGT8 phosphorylation has not been conducted. Phosphatase treatment caused a band shift of the UGT8 protein separated using Phos-tag SDS-PAGE, but the enzymatic activity of UGT8 was only slightly decreased, suggesting that phosphorylation of UGT8 is not critical for enzyme activity. Although the regulation of UGT8 enzymatic activity remains unclear, some biochemical analyses have demonstrated that mammalian UGTs form oligomers and that these interactions either positively or negatively affect UGT activity. For example, whilst the activity of UGT2B21 is upregulated by its interaction with UGTB22 *(**32**)*, UGT1A1 negatively controls the enzyme activity of UGT1A9 by interacting with it *(**33**)*. Interactome analysis of UGT8 may provide clues for a better understanding of the regulation of UGT8.

UGT8 was localized to the ER, which is consistent with a previous report *(**9**)*. The disruption of membrane lipid homeostasis by the elevation of saturated fatty acids in the ER, called membrane lipid saturation, causes ER stress *(**34**)*. The ER stress sensors IRE1 and PERK directly sense these stresses and activate the UPR *(**35*–*37**)*. The membrane lipid saturation-induced UPR was suppressed in UGT8 KO cells, as determined by PERK phosphorylation and downstream CHOP mRNA induction. The degree of lipid saturation of PC following SCD1 inhibitor treatment was comparable between WT and UGT8 KO cells. Considering that the MGDG content was far less than that of phospholipids (>0.1% of the amount of PC) but MGDG was more enriched in microsomal compartment compared to HexCer, we speculated that the UGT8-derived MGDG might be involved in the activation of PERK under membrane lipid saturation. Because PERK uses its transmembrane domain to sense membrane lipid saturation *(**36**)*, UGT8-derived MGDG may target the transmembrane domain of PERK to activate UPR signals.

In the present study, we characterized UGT8 as an MGDG-producing enzyme in HeLa cells. In addition, the MGDG content in mouse tissues was significantly decreased by UGT8 inhibitor treatment *(**7**)*. These results demonstrated that UGT8 is an MGDG synthase in mammals.

## Supplementary Material

Web_Material_mvae084
