# Supplementary figures and images for: Characterization of UGT8 as a monogalactosyl diacylglycerol synthase in mammals
Source: J Biochem. 2024 Dec 10;177(2):141–52. doi: 10.1093/jb/mvae084 (PMC11795506; doi:10.1093/jb/mvae084)

Figure S1

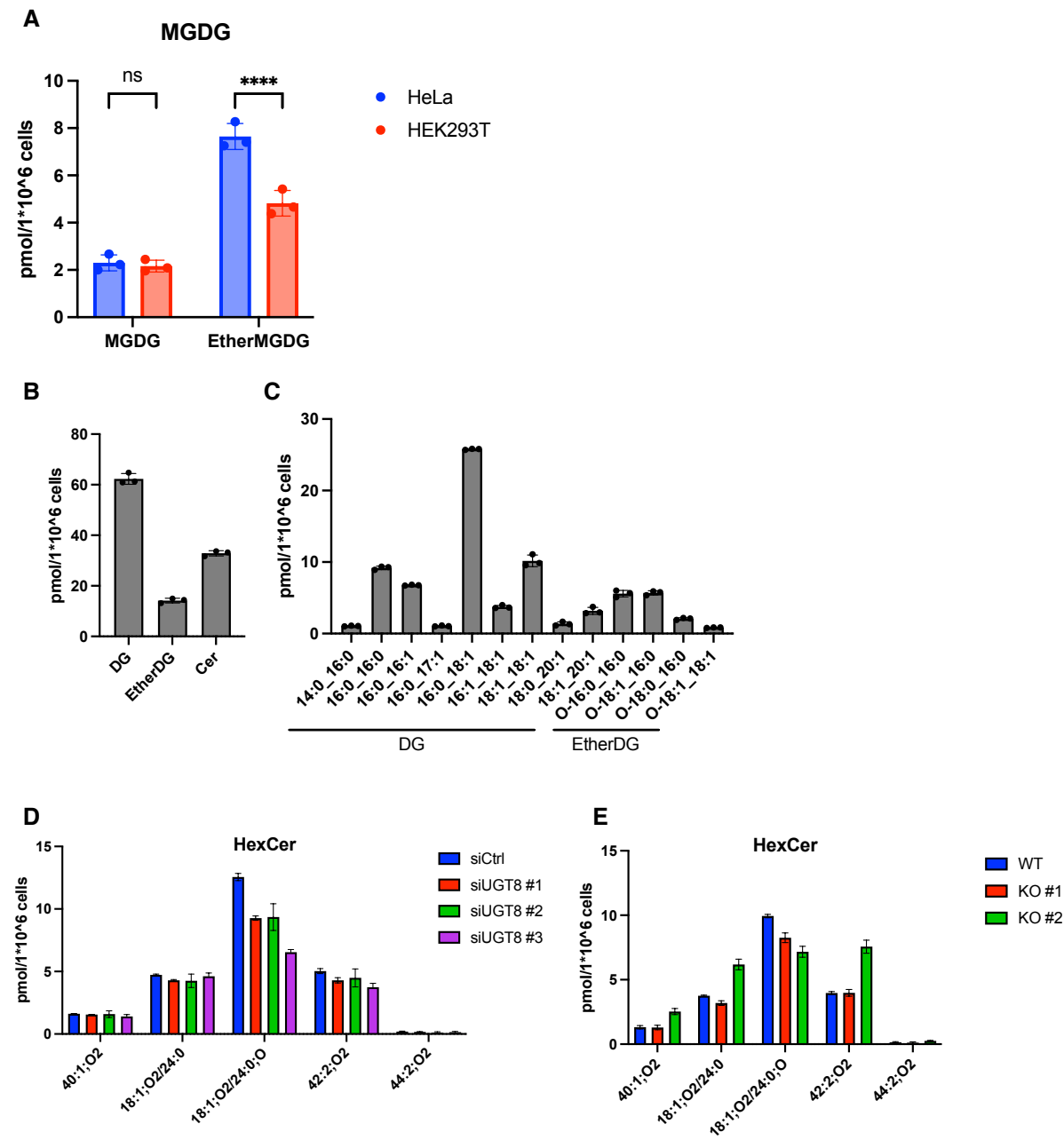

Supplement: Web_Material_mvae084 [file web_material_mvae084.zip › FigureS1.pdf]

Figure S2

A

MGalDG (10:0/10:0)/MGlcDG (10:0/10:0)

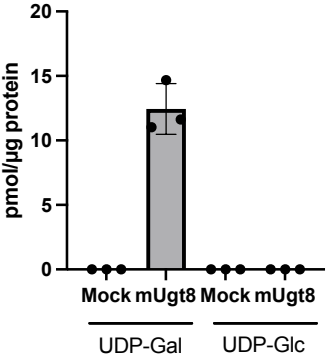

B

Gal C2Cer/Glc C2Cer

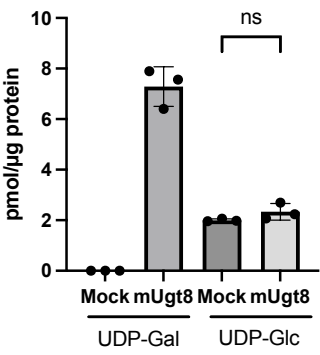

C

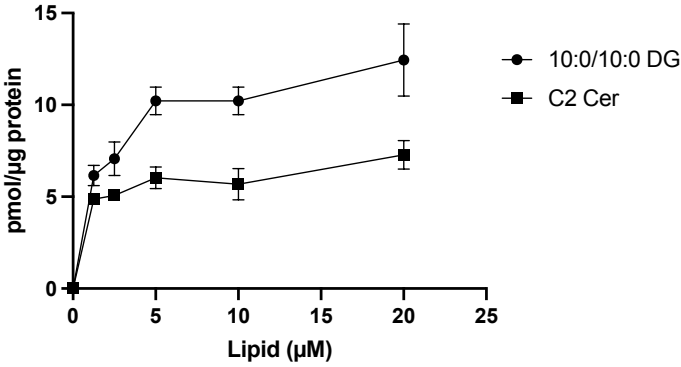

Supplement: Web_Material_mvae084 [file web_material_mvae084.zip › FigureS2.pdf]

**Figure S3**

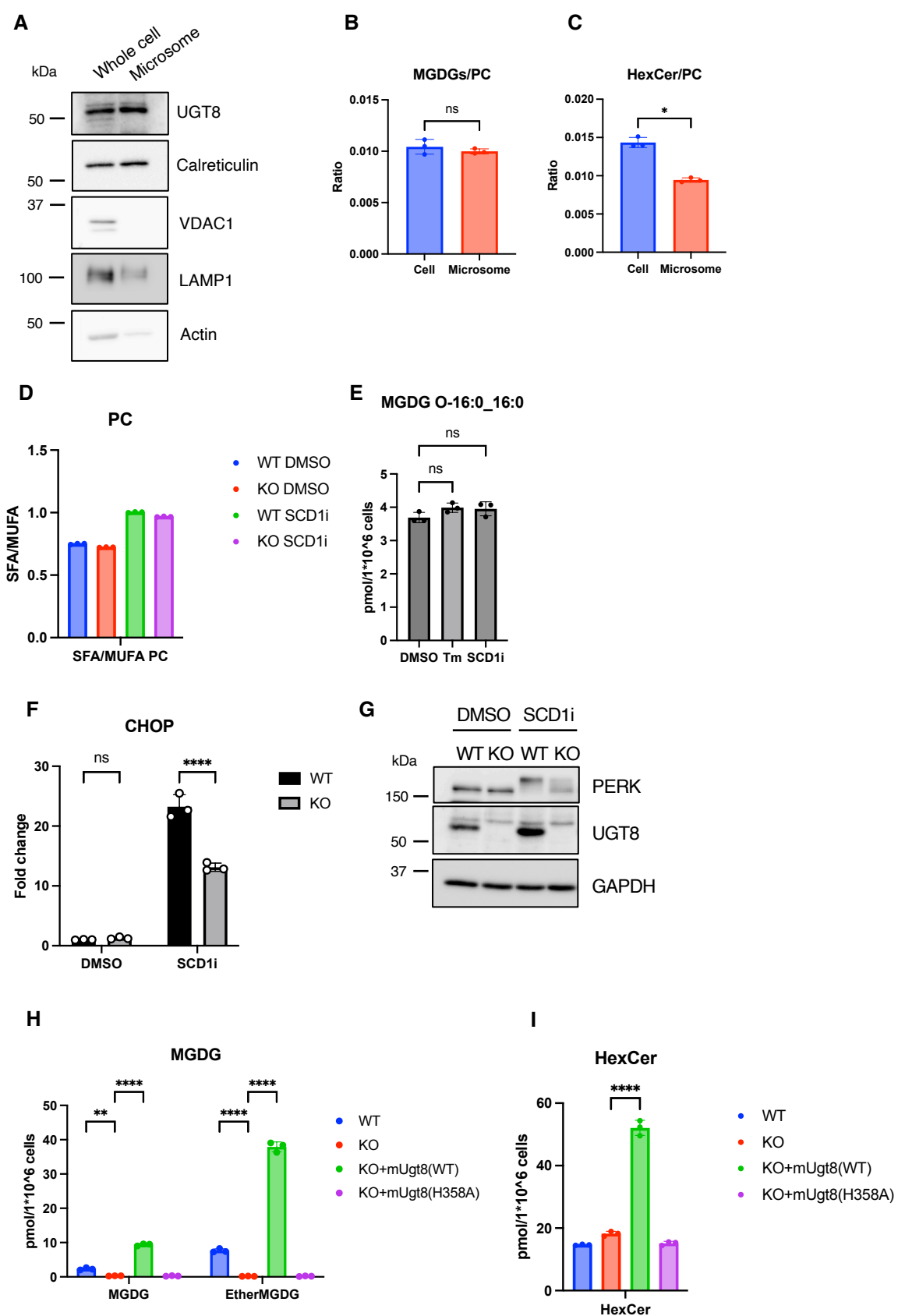

Supplement: Web_Material_mvae084 [file web_material_mvae084.zip › FigureS3.pdf]
